# Engaging Isolated and Underserved Older Adults in 4Ms Care: Age-Friendly Care, PA

**DOI:** 10.1093/geroni/igab046.1223

**Published:** 2021-12-17

**Authors:** Diane Berish, Terry Fulmer

## Abstract

Older adults, the largest segment of the US rural population, face significant disparities in health and healthcare compared to their non-rural peers, including more chronic health conditions, financial challenges, and social isolation. They have limited access to healthcare and social services for prevention, management and treatment of chronic conditions. Age-Friendly Care-PA, a partnership between Primary Health Network and Penn State College of Nursing, aims to reduce these disparities in care and services for rural older adults through co-designing their Geriatric Workforce Enhancement Program. Age-Friendly Health Systems, an initiative of the John A Hartford Foundation and the Institute for Healthcare Improvement, in partnership with the American Hospital Association and the Catholic Health Association of the United States, equips providers, older adults, and their care partners with the support necessary to address What Matters, Medication, Mentation, and Mobility. This symposium describes how the 4Ms are integrated into clinician training and competencies, older adult education, operations, care delivery, and quality improvement. Year two outcome data will be shared. Drs. Hupcey and Fick will provide an overview of the project and its reach. Dr. Berish will describe the process of engaging stakeholders in co-developing our 4M metrics and the data generated. Jenny Knecht, CRNP, will describe a pilot study to extend the reach and acceptability of telehealth to hard-to-reach older persons. Finally, Dr. Garrow will detail a new initiative focused on equity in care. Our discussant, Dr. Terry Fulmer will lead a discussion of this work as well as next steps and policy implications.

